# Light-Mediated Regulation of Leaf Senescence

**DOI:** 10.3390/ijms22073291

**Published:** 2021-03-24

**Authors:** Yasuhito Sakuraba

**Affiliations:** Plant Functional Biotechnology, Biotechnology Research Center, The University of Tokyo, Yayoi 1-1-1, Bunkyo-ku, Tokyo 113-8657, Japan; sakuraba0425@gmail.com

**Keywords:** leaf senescence, photosynthesis, reactive oxygen species, light signaling

## Abstract

Light is the primary regulator of various biological processes during the plant life cycle. Although plants utilize photosynthetically active radiation to generate chemical energy, they possess several photoreceptors that perceive light of specific wavelengths and then induce wavelength-specific responses. Light is also one of the key determinants of the initiation of leaf senescence, the last stage of leaf development. As the leaf photosynthetic activity decreases during the senescence phase, chloroplasts generate a variety of light-mediated retrograde signals to alter the expression of nuclear genes. On the other hand, phytochrome B (phyB)-mediated red-light signaling inhibits the initiation of leaf senescence by repressing the phytochrome interacting factor (PIF)-mediated transcriptional regulatory network involved in leaf senescence. In recent years, significant progress has been made in the field of leaf senescence to elucidate the role of light in the regulation of nuclear gene expression at the molecular level during the senescence phase. This review presents a summary of the current knowledge of the molecular mechanisms underlying light-mediated regulation of leaf senescence.

## 1. Introduction

Leaf senescence, the final stage of leaf development, is a highly controlled developmental process accompanied by massive transcriptional and metabolic changes that destabilize intracellular organelles and macromolecules and translocate nutrients into developing tissues and storage organs. Numerous studies conducted in the past two decades have greatly expanded our knowledge of the molecular mechanisms underlying the regulation of leaf senescence (reviewed in [1,2,3,4,5]). The initiation of leaf senescence is tightly controlled by endogenous factors, such as the state of phytohormones and other metabolites [5,6] and external stimuli such as drought, high salinity, high temperature, and pathogens [7,8,9,10]. Furthermore, a large number of transcription factors (TFs) involved in the regulation of leaf senescence has been identified in the model plant Arabidopsis (*Arabidopsis thaliana*) [5,11] and in other plant species [12], uncovering transcriptional regulatory networks that regulate leaf senescence.

Light is the foremost regulator of various biological processes in the plant life cycle. Light characteristics, such as wavelength, fluence rate, and photoperiod, greatly affect plant traits, including growth habit, floral induction, and plant productivity [13]. Plants transform light energy into chemical energy through the process of photosynthesis and also capture light energy using distinct sets of photoreceptors that perceive specific wavelengths of light through the process of light signaling [14]. Light is also a key determinant of the initiation of leaf senescence. In recent years, molecular mechanisms underlying photosynthesis- and light-signaling-mediated regulation of leaf senescence have been uncovered. This review highlights the significance of both photosynthesis and light signaling in the regulation of leaf senescence.

## 2. Regulation of Leaf Senescence by Light via Photosynthesis

Photosynthesis is the process that transforms light energy into chemical energy and thus forms the basis of all life on Earth, ranging from photosynthetic bacteria to higher plants. In plants, photosynthesis takes place in chloroplasts. Since chloroplasts contain approximately 80% of the total leaf nitrogen (N) [15], translocation of N compounds from chloroplasts in older leaves (source organs) to new developing tissues (sink organs) is one of the most crucial events during the process of leaf senescence. In addition, increased expression of senescence-associated genes (SAGs) is promoted by the decline in photosynthetic activity and photosynthesis-related gene expression [16]. Thus, the expression of SAGs during senescence is probably greatly affected by the status of photosynthesis, and the nucleus may obtain the information about the status of photosynthesis through signals produced in chloroplasts. The transport of such retrograde signals from chloroplasts to the nucleus to increase the expression of SAGs is still largely unknown, but several important clues have been found in recent studies.

### 2.1. Chloroplast-Produced Reactive Oxygen Species (ROS) Affect Leaf Senescence

**Figure 1:**
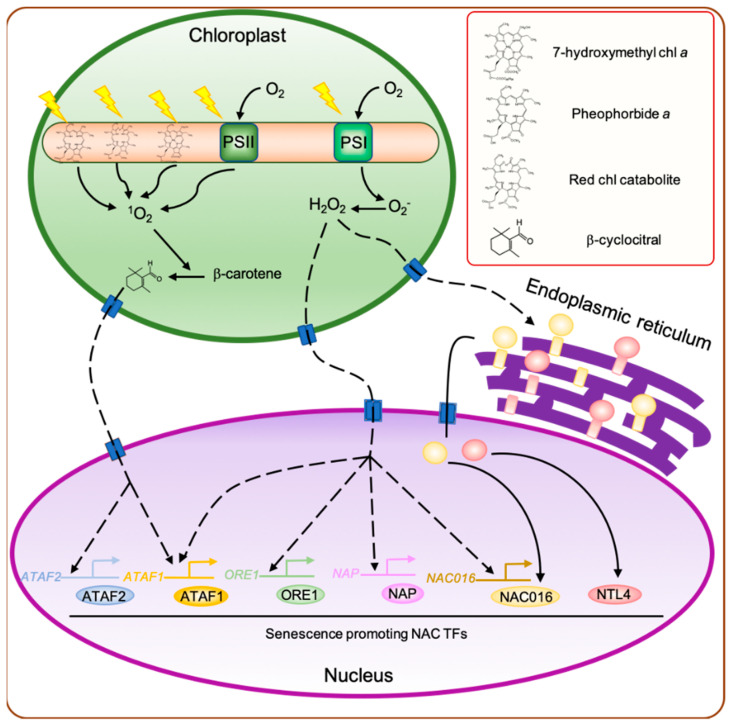
Regulatory network of leaf senescence induced by chloroplast-derived retrograde signaling. During the senescence phase, the abundance of photosystem proteins decreases significantly. Consequently, the captured light energy exceeds the amount needed by the remaining photosynthetic units, leading to the production of a large amount of O_2_^−^ by photosystem I (PSI) and ^1^O_2_ by PSII. In addition, the dismantling of light harvesting complex II (LHCII) proteins releases a large number of chlorophyll molecules and their degradation products, such as 7-hydroxymethyl chlorophyll *a*, pheophorbide *a*, and red chlorophyll catabolite, which act as photosensitizers, producing high amounts of ^1^O_2_ in the presence of light. The ^1^O_2_ generated in chloroplasts promotes the cleavage of β-carotene to produce β-cyclocitral, which then acts as a retrograde signal between the chloroplast and nucleus to activate the expression of ^1^O_2_-responsive genes, including senescence-associated *Arabidopsis thaliana* activating factor (ATAF) subfamily no apical meristem/*ATAF1,2*/cup-shaped cotyledon (NAC) genes, *ATAF1* and *ATAF2*. O_2_^−^ generated by PSI is rapidly dismutated into H_2_O_2_, which acts as a signaling molecule to alter the expression of H_2_O_2_-responsive genes, including *ATAF1*, *ORESARA1* (*ORE1*), *NAC-*like, activated by *AP3/PI* (*NAP*), and *ANAC016*. In addition, H_2_O_2_ generated in the chloroplasts also triggers the translocation of membrane-bound NAC transcription factors (TFs), such as ANAC016 and NAC with transmebrane motif 1-LIKE4 (NTL4), from the endoplasmic reticulum to the nucleus. Dashed arrowheads indicate the indirect activation of genes. chl, chlorophyll.

Reactive oxygen species (ROS) are highly reactive molecules generated by the reduction of ground-state molecular oxygen (^3^O_2_) through the acceptance of an electron pair. In plants, the major forms of ROS include superoxide anion (O_2_^−^), hydroxyl radical (·OH), hydrogen peroxide (H_2_O_2_), and singlet oxygen (^1^O_2_). Among these, O_2_^−^, ·OH, and H_2_O_2_ are produced in most subcellular compartments, while the highly reactive ^1^O_2_ is uniquely produced in chloroplasts, which are the primary source of ROS under light conditions [17]. ROS production in chloroplasts is tightly associated with light-dependent photosynthetic reactions. During photosynthesis, ^1^O_2_ is produced mainly within photosystem II (PSII) in thylakoid membranes because of the reaction between the triplet state of chlorophyll molecule and ^3^O_2_ [17]. On the other hand, the reduction of ^3^O_2_ by PSI generates O_2_^−^, which is immediately converted into H_2_O_2_ on the stromal side of the thylakoid membranes either spontaneously or through the action of superoxide dismutases (SODs) [18]. The production of ROS is drastically increased under unfavorable environmental conditions, such as excess light, drought, and high temperature [19]. In addition, it is well known that the production of ROS, especially H_2_O_2_, is significantly increased in leaves during senescence [20], which is accompanied by a significant reduction in the abundance of light harvesting complex II (LHCII) and other photosystem proteins. Consequently, the captured light energy is in excess for the remaining photosynthetic units, leading to the production of a large amount of ROS even under moderate light conditions. In addition, the dismantling of LHCII subunits releases a large number of chlorophyll molecules and their degradation products. It is well known that several chlorophyll biosynthesis intermediates, including protoporphyrin IX and protochlorophyllide, act as photosensitizers that produce high amounts of ^1^O_2_ under light conditions [21,22]. Similarly, several chlorophyll degradation products, such as 7-hydroxymethyl chlorophyll *a*, pheophorbide *a*, and red chlorophyll catabolite, also act as photosensitizers [23,24,25] (Figure 1).

For many years, it has been proposed that ROS generated in chloroplasts are involved in the promotion of leaf senescence (reviewed in [26,27]). Among these ROS, H_2_O_2_ plays a crucial role in the regulation of leaf senescence. Since H_2_O_2_ can easily cross cellular membranes [28], it can be transmitted as a signaling molecule from the chloroplast to the nucleus. Transcriptome analyses of Arabidopsis revealed that several genes upregulated during leaf senescence are also upregulated by H_2_O_2_ treatment [29,30]; for example, no apical meristem/ATAF1,2/cup-shaped cotyledon (NAC) TF-encoding genes, such as *Arabidopsis thaliana activating factor1 (ATAF1)/ANAC002* [31], *ANAC016* [32], *NAC-*like, activated by *AP3/PI (NAP)/ANAC029* [33], *JUNGBRUNNEN1 (JUB1)/ANAC042* [34], and *ORESARA1* (*ORE1*)*/ANAC092* [35], which have been characterized as key regulators of leaf senescence (Figure 1). Among these, *JUB1* acts as a negative regulator of leaf senescence, while the other four *NAC* genes promote leaf senescence.

H_2_O_2_ is also one of the triggers that induce the translocation of membrane-bound NAC TFs from the endoplasmic reticulum (ER) membrane to the nucleus. Several membrane-bound NAC TFs are activated by H_2_O_2_ generated in mitochondria under environmental stresses [36,37]. The induction of nuclear gene expression by treatment with methyl viologen, which accepts electrons from PSI via ferredoxin and leads to the production of a large amount of O_2_^−^ in chloroplasts, is strongly inhibited in the knockout mutant of *ANAC017*, an Arabidopsis membrane-bound NAC TF gene [38], indicating that H_2_O_2_ generated in chloroplasts is also important for the activation of membrane-bound NAC TFs. In Arabidopsis and rice (*Oryza sativa* L.), several membrane-bound NAC TFs including ANAC016 [32], NAC WITH TRANSMEMBRANE MOTIF 1-LIKE4 (NTL4)/ANAC053 [39], and ONAC054 [40] have been shown to promote the initiation of leaf senescence. Thus, H_2_O_2_ generated in chloroplasts during the senescence phase may modulate the gene expression partially via the activation of senescence-associated membrane-bound NAC TFs (Figure 1).

O_2_^−^ also modulates the expression of SAGs. In Arabidopsis seedlings treated with methyl viologen, an O_2_^−^-specific propagator, several genes encoding senescence-associated TFs including *ATAF1*, *WRKY6* [41], and *WRKY22* [42] were strongly upregulated [43]. WRKY6 and WRKY22 antagonistically regulate leaf senescence; WRKY6 acts as a negative regulator, while WRKY22 acts as an enhancer of leaf senescence. In contrast with H_2_O_2_, O_2_^−^ is a short-lived ROS and cannot cross the chloroplast membrane [44]. Thus, O_2_^−^ probably generated in chloroplasts cannot serve as a signaling molecule.

Compared with H_2_O_2_ and O_2_^−^, ^1^O_2_ is a highly reactive molecule and consequently a more potent oxidizing agent than the other ROS [45]. In leaf tissues, in particular, ^1^O_2_ is required for most of the lipid peroxidation reactions. Lipid peroxidation promotes the generation of free radicals, which accelerate senescence [46]. ^1^O_2_ also gives rise to a signal that affects the expression of nuclear genes, similar to the other two ROS. Op den Camp et al. (2003) investigated the effect of ^1^O_2_ on nuclear gene expression using Arabidopsis *flu* mutant, which shows greater accumulation of protochlorophyllide than the wild type. Since protochlorophyllide acts as a photosensitizer, the *flu* mutant generates a large amount of ^1^O_2_ when transferred from dark to light conditions [22]. In dark-grown *flu* mutant seedlings, incubation under light for 2 h significantly upregulated the expression of several stress-responsive genes including *ABSCISIC ACID* (*ABA*) *INSENSITIVE 1* (*ABI1*), which encodes a protein phosphatase that acts as a negative regulator of ABA signaling [47], and *1-AMINOCYCLOPROPANE-1-CARBOXYLATE OXIDASE 4* (*ACO4*), which encodes an ethylene biosynthesis enzyme [48]. In addition to their roles in abiotic stress response, ABA and ethylene are also known to promote leaf senescence [49]. Thus, ^1^O_2_ may affect the transcriptional regulatory networks of leaf senescence by modulating the expression of stress response and leaf-senescence-related genes.

### 2.2. State of Photosystem Proteins Determines the Initiation of Leaf Senescence

Several studies indicate that the state of photosystem proteins affects the initiation of leaf senescence. Chlorophyllide a oxygenase (CAO) is a chlorophyll biosynthesis enzyme that catalyzes two reactions: the conversion of chlorophyll *a* into 7-hydroxymethyl chlorophyll *a* and the subsequent transformation of 7-hydroxymethyl chlorophyll *a* into chlorophyll *b* [50]. The N-terminal domain of CAO proteins, which is required for their destabilization, is conserved among higher plants [51]. Transgenic Arabidopsis plants overexpressing N-terminal domain-truncated CAO overaccumulated chlorophyll *b* and stayed green during the senescence phase under both light and dark conditions [52]. Moreover, in these transgenic plants, the abundance of chlorophyll *b* in the antenna proteins of PSI and PSII increased significantly, and chlorophyll *b* was also incorporated into CP43, a PSII core protein, which otherwise binds only to chlorophyll *a* [53]. One of the possible explanations for the stay-green phenotype of these transgenic plants is the stabilization of photosystem proteins due to the increased incorporation of chlorophyll *b* in photosystem core complexes. Similar to the *CAO* overexpression lines, Arabidopsis and rice knockout mutants of *NON-YELLOW COLORING1* (*NYC1*), which encodes chlorophyll *b* reductase, also showed the stay-green phenotype with highly retaining of antenna proteins of photosystems, as well as the structure of grana thylakoid, during senescence [54,55]. Rice *delayed yellowing1* (*dye1*) mutants, in which the pigment-binding function of one of the LHCI subunits (Lhca4) was impaired, exhibited prolonged greenness during the senescence phase [56]. In *dye1* mutant plants, although the accumulation of other Lhca proteins (Lhca1, Lhca2, and Lhca3) was similar to that in the wild type, Lhcb1 (a subunit of LHCII) showed high accumulation [56]. Since the chlorophyll *b* content of the *dye1* mutant increased because of the increase in the chlorophyll *b*-binding capacity of LHCII proteins, this result also supports the hypothesis that the abundance of chlorophyll *b* is positively correlated with the stability of PSI and PSII during senescence. Additionally, a large number of SAGs were differentially expressed between *CAO*-overexpressing and wild-type Arabidopsis plants [52]. Thus, the stabilization of photosystem proteins, partially by chlorophyll *b*, is one of the key factors affecting the abundance of signaling molecules required for the induction of leaf-senescence-related transcriptional-regulatory networks.

### 2.3. Potential Role of Chlorophyll and Caroteinoid Degradation Products as Retrograde Signaling Molecules in the Regulation of Leaf Senescence

Chlorophyll biosynthesis and degradation intermediates have the potential to produce ROS as photosensitizers, as described above. However, according to several other studies, some chlorophyll intermediates can mediate retrograde signaling from the chloroplasts to the nucleus. It has been shown that Mg-protoporphyrin IX (Mg-proto IX) can act as a negative regulator of nuclear genes associated with photosynthesis. Exogenous application of Mg-proto IX to Arabidopsis protoplasts has been shown to greatly inhibit the expression of Lhcb [57]. In addition, Mg-proto IX can bind to heat-shock protein 90 (HSP90) to decrease its ATPase activity, which subsequently decreases the expression of genes associated with photosynthesis, probably via the regulation of LONG HYPOCOTYL5 (HY5) [58]. Considering the function of Mg-proto IX, it is also probable that chlorophyll degradation intermediates, such as 7-hydroxymethyl chlorophyll *a*, pheophytin *a*, and pheophorbide *a*, act as signaling molecules. Indeed, several genes associated with chlorophyll degradation were upregulated in transgenic Arabidopsis plants overexpressing *PHEOPYTINASE* (*PPH*) [59], which encodes a chlorophyll degradation enzyme that catalyzes the conversion of pheophytin *a* into pheophorbide *a* [60]. Similarly, STAY-GREEN1 (SGR1), a chlorophyll degradation enzyme that converts chlorophyll *a* into pheophytin *a* [61], also affects the expression of genes associated with leaf senescence. For example, when *SGR1* was overexpressed via the dexamethasone (DEX)-inducible system, genes associated with chlorophyll degradation, such as *PPH*, *SGR2* [61], *NYC1* [55], and *PAO* [24], and other SAGs, such as *ORE1*, were significantly upregulated [62]. These results indicate that the accumulation of chlorophyll degradation intermediates or changes in the activity of chlorophyll degradation enzymes may lead to retrograde signaling between the chloroplast and nucleus to activate the transcriptional regulatory networks involved in leaf senescence. The other possibility is that chlorophyll degradation intermediates themselves act as signaling molecules.

Ramel et al. (2012) revealed that in Arabidopsis, high light-induced ^1^O_2_ promotes the cleavage of β-carotene, and the resulting breakdown products including β-cyclocitral mediate ^1^O_2_-related retrograde signaling to modulate the expression of genes associated with photooxidative stress [63]. Among these genes, four *NAC* genes, *ATAF1*, *ANAC032*, *ATAF2/ANAC081*, and *ANAC102*, which encode a senescence-promoting NAC TF [31,64], are strongly upregulated by both β-cyclocitral and high light intensity [65]. Given the elevated level of ^1^O_2_ in chloroplasts during senescence phase, β-cyclocitral-mediated retrograde signaling may play a role in the regulation of leaf senescence

## 3. Regulation of Leaf Senescence by Light Signaling

In recent years, numerous studies have been conducted to understand the relationship between light signaling and leaf senescence, and their results have expanded our knowledge of the role of light signaling in the initiation of leaf senescence. In Section 3.1, the current knowledge of the functions of plant photoreceptors is briefly summarized as a background to the light-signaling-mediated regulation of leaf senescence.

### 3.1. Role of Photoreceptors in Plants

Plant growth and development are optimized through the perception of light across the spectral range of 300–800 nm [14]. Within this spectrum, light wavelengths ranging from 400 to 700 nm, which together comprise photosynthetically active radiation [66], are used for photosynthesis. On the other hand, plants have distinct sets of photoreceptors that together enable the perception of light across a broad spectrum, ranging from ultraviolet-B (UV-B, 280–315 nm) to far-red (700–800 nm), and the induction of wavelength-specific responses. Cryptochrome is one of the blue-light receptors that perceives blue light (approximately 380–500 nm) and UV-A light (approximately 320–380 nm) and is involved in the regulation of various biological phenomenon including the circadian clock, flowering, and photomorphogenesis [67]. Another type of blue-light photoreceptor, phototropin, regulates chloroplast movement, stomatal opening, and phototropic curvature [67]. Furthermore, three LOV/F-box/Kelch-repeat proteins, namely ZEITLUPE (ZTL), FLAVIN-BINDING KELCH REPEAT, F-BOX1 (FKF1), and LOV KELCH REPEAT PROTEIN2 (LKP2), have also been identified as blue-light receptors and are involved in the regulation of circadian rhythms and photoperiodic flowering [66]. UV RESISTANCE LOCUS 8 (UVR8) perceives UV-B light and regulates UV-B-light-specific responses such as cotyledon opening and flavonoid synthesis [68]. Phytochrome is the sole photoreactor that perceives red light (approximately 600–700 nm) and far-red light and regulates a variety of physiological processes including flowering, seed germination, seedling de-etiolation, and shade avoidance response [69]. The model plant Arabidopsis contains five phytochromes, namely phyA, phyB, phyC, phyD, and phyE [70]. These phytochromes can be divided into two groups, according to their primary photosensory activities and physiological roles. Among these, phyB–phyE are involved in the red/far-red low-fluence response via the reversible transition between the red-light-absorbing (biologically inactive) Pr form and the far-red-light absorbing (biological active) Pfr form; only the Pfr form can move from the cytosol to the nucleus to activate the red-light signaling pathway. Among these four phytochromes, the abundance of phyB is far greater than those of the other three phytochromes. Unlike these four phytochromes, phyA plays an important in the far-red-light response [71]. Thus, these photoreceptors differentially regulate a variety of light-responsive events.

When these photoreceptors are activated by a specific wavelength of light, they move into the nucleus and modulate the activity of TFs specific for each light signaling cascade. For instance, under red-light illumination, the biologically active Pfr form moves into the nucleus, and it interacts with phytochrome interacting factors (PIFs), which are helix–loop–helix (bHLH) TFs and acts as a negative regulator of red light signaling [72]. Similarly, photoactivated cryptochromes move into the nucleus to interact with CRYPTOCHROME-INTERACTING BASIC-HELIX-LOOP-HELIX (CIB) TFs to negatively regulate blue-light signaling [73]. A basic leucine zipper (bZIP)-type transcription factor, HY5, and its functional homolog, HY5 HOMOLOG (HYH), are also key regulators of light signaling. The activity of HY5 and HYH is regulated by the CONSTITUTIVELY PHOTOMORPHOGENIC 1 (COP1)–SUPPRESSOR OF PHYA1 (SPA1) complex in the nucleus via the E3 ligase activity of COP1 [74]. Importantly, three different light components (red light, far-red light, and blue light) affect COP1–SPA1-mediated degradation of HY5 [74]. Thus, HY5 and HYH act as key regulators of light signaling by integrating the information from multiple light signaling pathways. Recent studies revealed some of the molecular mechanisms underlying red-, far-red-, and blue-light-mediated regulation of leaf senescence.

### 3.2. Red-Light Signaling-Mediated Regulation of Leaf Senescence in the Model Plant Arabidopsis

Red light has long been considered to delay leaf senescence. In 1970s and 1980s, the effect of red light on the initiation of leaf senescence was extensively investigated and was shown to retard chlorophyll degradation in the leaves of barley (*Hordeum vulgare* L.) [75,76], tomato (*Solanum lycopersicum* L.) [77], cucumber (*Cucumis sativus* L.) [77], and mustard (*Sinapis alba* L.) [78], indicating that red light delays the initiation of leaf senescence.

**Figure 2:**
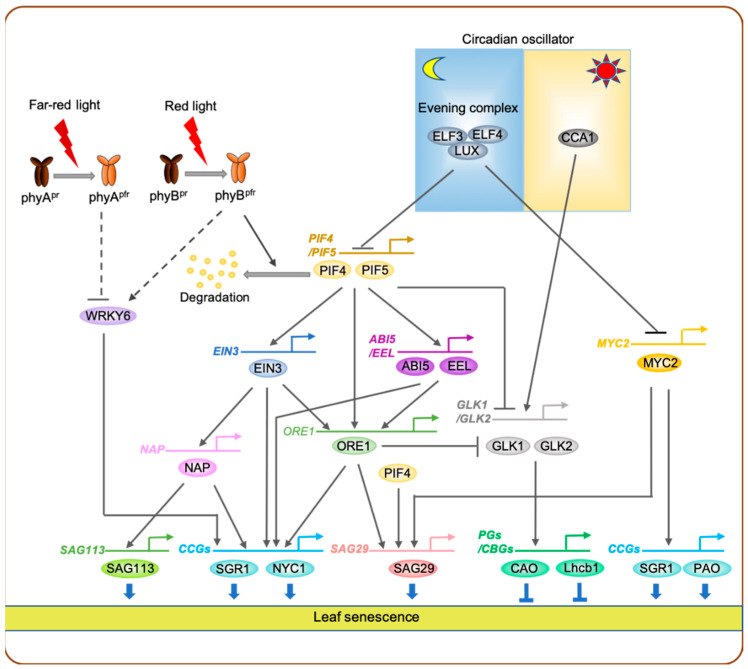
Transcriptional regulatory network of leaf senescence regulated by light signaling. Under red light, PIF4 and PIF5 are degraded through a phyB-mediated proteasomal degradation pathway, leading to the inactivation of PIF-dependent promotion of leaf senescence. In darkness, however, phyB exists in the inactive Pr state, which allows PIF4 and PIF5 to activate the expression of *ETHYLENE INSENSITIVE3* (*EIN3*), *ABSCISIC ACID INSENSITIVE 5* (*ABI5*), and *ENHANCED EM LEVEL* (*EEL*). Then PIFs, together with EIN3, ABI5, and EEL, form a feedforward loop to activate the expression of *ORE1*. Among the proteins that act downstream of PIF4 and PIF5, ORE1 is involved in feedforward loops that activate genes, encoding chlorophyll degradation enzymes, repressing *GOLDEN2-LIKE* (*GLK*) genes, which encode transcriptional activators of photosynthesis and chlorophyll biosynthesis-related genes. In the circadian oscillator, the EARLY FLOWERING3 (ELF3)–ELF4–LUX evening complex (EC) represses the expression of *PIF4* and *PIF5* as well as that of *MYC2*, which encodes a key TF involved in jasmonate signaling, while CIRCADIAN CLOCK ASSOCIATED 1 (CCA1), a core component of the circadian oscillator, activates the expression of *GLK* genes and represses the expression of *ORE1*. *WRKY6*, which encodes a senescence-associated WRKY TF, is negatively and positively regulated by phyA- and phyB-mediated signaling pathways, respectively. Solid lines indicate direct regulation, while dashed lines indicate indirect regulation. CCGs, chlorophyll catabolic genes; PGs, photosynthesis genes; CBGs, chlorophyll biosynthetic genes.

In contrast to previous research, molecular mechanisms underlying red-light signaling-mediated promotion of leaf senescence have been revealed in the last decade, especially in Arabidopsis. During dark-induced leaf senescence, yellowing of cotyledons in the *A. thaliana* ecotype Columbia (Col-0; wild type) was strongly inhibited by intermittent red-light pulse; however, this delayed leaf senescence phenotype was diminished by far-red-light pulse [79]. In addition, *phyB* mutants exhibited insensitivity to red-light pulse-induced retardation of leaf yellowing, indicating that phyB acts as the main photoreceptor for red-light signaling-mediated promotion of leaf senescence [79]. Among the four PIFs (PIF1, PIF3, PIF4, and PIF5) that are regulated by the active Pfr form of phyB, PIF4 and PIF5 positively regulate both age-dependent and dark-induced leaf senescence [79] (Figure 2).

A number of downstream target genes of PIF4 and PIF5 involved in leaf senescence pathways have been identified to date. During dark-induced leaf senescence, PIF4 and PIF5 directly activate the transcription of *ORE1*, which encodes a senescence-associated NAC TF and is considered a key promoter of leaf senescence in Arabidopsis [35]. Furthermore, both PIF4 and PIF5 mediate the signaling of two senescence-promoting phytohormones, ethylene and ABA [80,81], by directly activating the expression of *ETHYLENE INSENSITIVE 3* (*EIN3*), which encodes a key ethylene signaling TF [82], and *ABI5* and *ENHANCED EM LEVEL* (*EEL*), which are two sister genes encoding bZIP-type ABA signaling TFs [83]. Moreover, EIN3, ABI5, and EEL were also shown to directly activate *ORE1* expression. Thus, PIF4, PIF5, EIN3, ABI5, and EEL form multiple coherent feedforward loops to activate *ORE1* expression [79] (Figure 2). Both PIF4 and PIF5 were also shown to directly activate the transcription of genes encoding chlorophyll catabolic enzymes. PIF4 and PIF5 directly bind to the promoter of *SGR1* to activate its expression [84,85]. In addition, PIF5 also activates the transcription of *NYC1* by binding to its promoter [85]. Moreover, ABI5, EEL, ORE1, and EIN3 also directly activate *SGR1* and *NYC1* expression [79,86]. Thus, PIF4, PIF5, and their downstream senescence-associated TFs form coherent feedforward loops to activate genes associated with chlorophyll degradation during leaf senescence (Figure 2). Such coherent feedforward loops regulate many biological processes in plants, such as chlorophyll biosynthesis, floral induction, and leaf senescence [35,87,88], and are thought to increase the robustness of transcriptional regulatory networks [89].

*GOLDEN2-LIKE 1* (*GLK1*) and *GLK2*, which encode GARP-type MYC TFs that directly activate genes encoding photosystem proteins and chlorophyll biosynthesis enzymes [90], have also been identified as downstream targets of PIF TFs during leaf senescence. In darkness, PIF4 and PIF5 protein levels increase because of the reduction in the activity of phytochromes [79], leading to decreased expression levels of *GLKs* [84]. Consequently, phenomena such as photosynthesis and chlorophyll biosynthesis are downregulated during dark-induced leaf senescence. PIF TFs have also been shown to directly activate or repress other known SAGs, including *JUB1*/*ANAC042* [91] and *SENESCENCE ASSOCIATED GENE 29* (*SAG29*) [92], although the significance of these transcriptional cascades in leaf senescence has not yet been validated. Taken together, in Arabidopsis, phyB-mediated red-light signaling inhibits the initiation of leaf senescence via multiple regulatory cascades composed of many SAGs (Figure 2).

Recently, in Arabidopsis, Kim et al. (2019) reported that phyB and PIFs also mediate high-temperature-induced leaf senescence. While the light-activated Pfr form of phyB is inactivated in darkness at 20 °C, it is inactivated more rapidly at 28 °C [93]. This inactivation of phyB at high temperature leads to an increase in PIF4 protein levels, which activates *ORE1* expression directly or indirectly through ABA and ethylene signaling, thus promoting leaf senescence at high temperature [93]. Thus, the phyB–PIF signaling module also integrates temperature signaling to regulate leaf senescence.

### 3.3. Red-Light Signaling-Mediated Regulation of Leaf Senescence in Crops

While the mechanisms underlying phyB-mediated red-light signaling in the regulation of leaf senescence have been intensively studied in Arabidopsis, information available in other plant species is limited. Piao et al. (2015) reported that similar to Arabidopsis *phyB* mutants, rice *phyB* (*OsphyB*) T-DNA insertion knockout mutants exhibited accelerated leaf yellowing during dark-induced senescence [94], indicating that Arabidopsis and rice phyB homologs exhibit similar roles in the regulation of leaf senescence. Among the PIF TFs in rice, PIF-LIKE1 (OsPIL1; also known as OsPIL13) shows high sequence similarity with Arabidopsis PIF4 and PIF5 [95]. However, unlike the delayed leaf yellowing phenotype of Arabidopsis *pif4* and *pif5* mutants, leaves of the *ospil1* knockout mutant turned yellow much faster than wild-type leaves [96], suggesting that OsPIL1 acts as a negative regulator of leaf senescence in rice. Interestingly, transgenic Arabidopsis plants overexpressing *OsPIL1* exhibited an accelerated leaf yellowing phenotype during dark-induced leaf senescence, similar to Arabidopsis *pif4* and *pif5* mutants [96], indicating that OsPIL1 and Arabidopsis PIF4 and PIF5 play similar roles in the regulation of leaf senescence, at least in Arabidopsis. Probably, the differences in their downstream cascades between Arabidopsis and rice modified their effects on the induction of leaf senescence. In a similar case, Arabidopsis EARLY FLOWERING3 (ELF3) acts as a negative regulator of leaf senescence [79], while OsELF3 acts as a positive regulator since *oself3* knockout mutants exhibited delayed leaf yellowing during both natural and dark-induced leaf senescence [97], in contrast with Arabidopsis *elf3* mutant [79]. Since ELF3 downregulates *PIF4* and *PIF5* expression in Arabidopsis [79], *OsPIL1* may be one of the key downstream target genes of OsELF3 in the leaf senescence pathway, and may explain, at least partially, the opposite effects of ELF3 homologs on leaf senescence initiation in Arabidopsis and rice.

In tomato, SlPIF4 was found to exhibit similar functions as Arabidopsis PIF4 and PIF5 and has been shown to affect plant size, flowering, auxin level, and thermomorphogenesis [98]. In addition, silencing of *SlPIF4* via RNA interference (RNAi) delayed leaf yellowing during the senescence phase and downregulated several SAGs, such as *SlSGR1*, *SlSAG12*, and *SlORE1* [98]. These findings suggest that SlPIF4 promotes leaf senescence by modulating downstream transcriptional cascades, similar to Arabidopsis PIF4 and PIF5. Moreover, similar to transgenic Arabidopsis lines overexpressing *OsPIL1*, those overexpressing maize *PIF4* (*ZmPIF4*) also exhibited accelerated leaf-yellowing phenotype during the senescence phase [99]. Phenotypic characterization of *ZmPIF4* overexpression lines and *zmpif4* knockout mutants is necessary to further understand the role of ZmPIF4 in the regulation of leaf senescence.

### 3.4. Red-Light Signaling-Mediated Regulation of Leaf Senescence Induced by Nutrient Deficiency

While phyB-mediated red-light signaling regulates dark-induced leaf senescence, as described above, this mechanism has also been shown to affect leaf senescence under light conditions. Detached leaves of *osphyB* knockout mutants turned yellow much faster than wild-type leaves when they were incubated in nutrient-free liquid medium under continuous light conditions [94]. In addition, the early-yellowing phenotype of *osphyB* mutant leaves was recovered by the supplementation of N compounds, such as potassium nitrate (KNO_3_) and ammonium nitrate (NH_4_NO_3_) [94], indicating that N status is important for OsphyB-mediated regulation of leaf senescence under light conditions.

N is one of the key macronutrients essential for plant growth and development [100]. The effects of light on N acquisition and use have been widely studied (summarized in [101]). In several plant species, including Arabidopsis, maize, soybean (*Glycine max* L.), sunflower (*Helianthus annuus* L.), tobacco (*Nicotiana benthamiana*), and wheat (*Triticum aestivum* L.), plant roots showed increased uptake of ^15^N-labeled nitrate (^15^NO_3_^−^) and nitrite (^15^NO_2_^−^) upon exposure to light [102,103]. Additionally, exogenous application of sugar to plant roots greatly increased NO_3_^−^ and ammonium (NH_4_^+^) uptake [103,104]. These data suggest that the positive effects of light on the uptake of nutrients, including N compounds, are linked in part to the increase in carbohydrate supply from leaves to roots that result from the increase in photosynthesis caused by the increase in light availability. On the other hand, several reports implied that light signaling promotes the uptake of N as well as other nutrients. In Arabidopsis seedlings, the expression of ammonium transporter genes (*AMT1;1*, *AMT1;2*, and *AMT2;1*) was enhanced upon irradiation with red light [105]. HY5, a key transcription factor that positively regulates red-light signaling downstream of phyB was shown to bind to and activate the promoter of *NITRATE TRANSPORTER2.1* (*NRT2.1*), encoding a high-affinity nitrate transporter [106]. Furthermore, genome-wide chromatin immunoprecipitation sequencing (ChIP-seq) analysis indicated that HY5 directly binds to the promoters of genes associated with the uptake and assimilation of N and other nutrients [107]. Another ChIP-seq analysis showed that PIF4 also directly binds to the promoters of genes associated with nutrient uptake and assimilation, such as *NRT1.1* and *NRT1.2* as well as *NIA2*, which encodes a nitrate reductase [108]. Moreover, nitrate reductase activity was significantly higher in *pif4* mutants than in the wild type, especially under red light [109], indicating that HY5 and PIF4 TFs positively or negatively regulate N uptake and assimilation under the regulation of phyB-mediated red-light signaling. It was recently shown that phyB/PIF4/PIF5/HY5-mediated red-light signaling is also involved in the promotion of phosphate (PO_4_^3−^) uptake in roots by increasing the expression of phosphate transporter genes, including *PHOSPHATE TRANSPORTER1;1* (*PHT1;1*) [110]. Thus, phyB-mediated red-light signaling promotes the uptake and utilization of some nutrients, including N and phosphorus (P), which negatively affects the initiation of leaf senescence.

### 3.5. Far-Red-Light Signaling-Mediated Regulation of Leaf Senescence

In contrast to red light, far-red light accelerates leaf senescence. Under shade (low red: far-red ratio), leaves exhibit accelerated leaf yellowing [111]. In addition, far-red light illumination decreases chlorophyll pigments and/or promotes leaf yellowing in various plant species, including liverwort (*Marchantia polymorpha*), cucumber, rice, and Pak choi (*Brassica rapa* ssp. *chinensis*) [77,112,113,114].

Far-red-light illumination diminishes red-light signaling by changing the active Pfr form to the inactive Pr form [69], which may, at least partially, contribute to the promotion of leaf yellowing. Indeed, the intermittent red-light pulse treatment strongly inhibited the increase in PIF4 and PIF5 protein levels in darkness, while pulses of red light followed by far-red light diminished this inhibitory effect [79]. In Pak choi leaves, far-red-light illumination enhanced, whereas red-light illumination reduced the expression of key SAGs, including *EIN3* [114].

Lim et al. (2019) showed that under far-red light-enriched conditions, leaves of the Arabidopsis *phyA* loss-of-function mutant (*phyA-211*) turned yellow much faster than those of wild-type plant, while the *phyB* loss-of-function mutant (*phyB-9*) exhibited delayed leaf yellowing [115]. Under far-red light illumination, the expression of *WRKY6* [116], was upregulated in *phyA* loss-of-function mutant but downregulated in *phyB* loss-of-function mutant [115]. Thus, phyA and phyB are involved in fine-tuning the far-red light-mediated promotion of leaf senescence by antagonistically regulating the expression of far-red light-inducible SAGs including *WRKY6* (Figure 2).

FAR-RED IMPAIRED RESPONSE 1 (FAR1) and its closest homolog FAR-RED ELONGATED HYPOCOTYL3 (FHY3), which are transposase-derived TFs, play key roles in phyA-mediated far-red-light signaling pathways [117,118]. It was recently shown that FHY3 acts as a negative regulator of age-dependent and light signaling-mediated leaf senescence by directly repressing the transcription of *WRKY28*, which encodes a senescence-promoting WRKY TF [119]. Both *fhy3* knockout mutant and *WRKY28* overexpression lines exhibited early leaf yellowing under light conditions with high red: far-red ratio [119], suggesting that the FHY3–WRKY28 regulatory module plays an important role in the inhibition of leaf senescence under high red: far-red ratio.

### 3.6. Blue-Light Signaling-Mediated Regulation of Leaf Senescence

The effects of blue light on the initiation of leaf senescence appear to be much smaller than those of red light in some plant species. Postharvest senescence of broccoli (*Brassica oleracea* L.) was delayed by red-light-emitting diodes (LEDs) but was not affected by blue LEDs [120]. Similarly, red LEDs inhibited leaf yellowing in Pak choi, while the inhibitory effect of blue LEDs was much smaller [114].

The role of blue-light signaling on the initiation of leaf senescence is not yet well understood. CRYPTOCHROME 1 (CRY1) and (CRY2) are blue light photoreceptors that regulate a variety of blue-light-mediated biological processes [67]. The *cry1 cry2* double mutant of Arabidopsis turned yellow, similar to the wild type, during dark-induced leaf senescence [79], indicating that cryptochrome-mediated blue light signaling plays little or role in dark-induced leaf senescence in Arabidopsis. On the other hand, in soybean, *CRY2a* acts as a negative regulator of leaf senescence; transgenic plants overexpressing *CRY2a* exhibited delayed leaf yellowing, whereas *CRY2a* RNAi plants exhibited accelerated-leaf-yellowing phenotype during the senescence phase [121]. In addition, CIB1 acts as an enhancer of leaf senescence [121]. Under blue light, CRY2a undergoes light-specific interaction with CIB1, which decreases the *WRKY53b* promoter-binding affinity of CIB1 [121]. Thus, CRY2a- and CIB1-mediated blue light signaling inhibits the initiation of leaf senescence in soybean. This shows that the effects of cryptochrome-mediated blue-light signaling on the initiation of leaf senescence varies among plant species.

In Arabidopsis, CRY1 directly interacts with PIF4 in a blue-light-dependent manner to repress its gene transcription in high-temperature-mediated hypocotyl elongation [122]. Thus, it is possible that CRY1-mediated blue-light signaling affects the initiation of leaf senescence under specific environmental stresses, such as high temperature, through the regulation of PIF4 activity, although this effect may be small compared with the effect of red light.

### 3.7. Connection between Circadian Rhythm and Light Signaling during Leaf Senescence

Recent studies show that circadian rhythm plays an important role in the regulation of leaf senescence. In Arabidopsis, the evening complex (EC), composed of ELF3, ELF4, and LUX APRHYTHMO (LUX), negatively regulates jasmonate (JA)-induced leaf senescence [123]. This EC was shown to directly bind to the promoter of *MYC2*, which encodes a key JA response regulator [124], and represses its expression, leading to the increase of expression of a number of JA-inducible genes [123]. In addition, plants carrying mutations in *ELF3*, *ELF4*, and *LUX* exhibited accelerated leaf yellowing during natural senescence and dark-induced leaf senescence [125] (Figure 2). In a diurnal rhythm, EC represses the expression of *PIF4* and *PIF5* early during the subjective night [126]. This EC–PIF4/PIF5 regulatory cascade is also important in dark-induced leaf senescence; the expression levels of *PIF4* and *PIF5* were upregulated in *elf3* mutants but downregulated in *ELF3* overexpressors after a few days of dark incubation [79].

It has recently been reported that CIRCADIAN CLOCK ASSOCIATED 1 (CCA1), one of the core components of the circadian oscillator, negatively regulates leaf senescence by directly repressing the expression of *ORE1*, which acts downstream of PIF4 and PIF5 in the red-light signaling-mediated leaf senescence pathway [79], while directly activating the expression of *GLKs* [127] (Figure 2). PSEUDO-RESPONSE REGULATOR 9 (PRR9), which is another component of the circadian oscillator, promotes leaf senescence by directly enhancing *ORE1* expression [125]. Thus, several components of the circadian oscillator, including ELF3, ELF4, LUX, CCA1, and PRR9 affect the expression of genes associated with red-light signaling-mediated leaf senescence, indicating that the circadian rhythm is closely linked with the transcriptional regulatory network of light signaling-mediated leaf senescence.

## 4. Conclusions and Perspectives

In conclusion, both photosynthesis and light signaling regulate the initiation and progress of leaf senescence through complex regulatory networks involving a large number of genes. Evidence generated in recent years demonstrates the close association of photosynthesis and light signaling with signaling responses in a variety of biological processes. Thus, the regulatory networks controlling photosynthesis- and light signaling-mediated leaf senescence may be much more intricate than what is currently known. For instance, light signaling-mediated shoot growth is regulated by several phytohormones including auxin, cytokinin, ethylene, and gibberellin [128]. Additionally, light signaling-mediated chlorophyll biosynthesis is also regulated by cytokinin, ethylene, and gibberellin [129]. Since these phytohormones are also known to positively or negatively regulate the initiation and progress of leaf senescence [130], it is possible that light signaling regulates leaf senescence, at least partially, via the signaling cascades of these phytohormones.

Leaf senescence is regulated in an age-dependent manner as well as by a variety of environmental factors including light [5]. As introduced in this review, plants utilize light for photosynthesis and light signaling to regulate the initiation of leaf senescence. Striking a balance between signaling cascades may be important, depending on various environmental conditions.

It is also important to understand the effect of the crosstalk between photosynthesis and light signaling pathways on the initiation and progress of leaf senescence. Expression levels of several key SAGs, such as *ORE1*, are affected by ROS generated by the alteration of photosynthetic capacity and phyB-mediated red-light signaling [30,79]. Thus, it is possible that photosynthesis-mediated and light signaling-mediated responses on the regulation of leaf senescence are integrated by some common components among their signaling cascades. Although this interplay between the two signaling responses is not yet fully understood, one of potential candidates that connects that two response pathways is HY5. As described in Section 3.1, HY5 plays important roles in the induction of genes associated with red-, far-red-, and blue-light signaling [101]. Moreover, HY5 is also involved in the regulation of genes associated with photosynthesis through Mg-proto IX-mediated chloroplast–nucleus retrograde signaling [58]. Furthermore, HY5 has also been shown to directly bind to the promoters of photosynthesis-associated genes to regulate their expression [107]. Thus, HY5 not only plays a critical role in both photosynthesis- and light signaling-mediated regulation of leaf senescence separately but also acts as a key player in the crosstalk between these two pathways.
